# Supplementary material for: Mechanisms governing the pioneering and redistribution capabilities of the non-classical pioneer PU.1
Source: Nat Commun. 2020 Jan 21;11:402. doi: 10.1038/s41467-019-13960-2 (PMC6972792; doi:10.1038/s41467-019-13960-2)
Supplement: Supplementary file 7 — Source data [file 41467_2019_13960_MOESM7_ESM.zip › Source_Data/FigureS2/FigureS2B_MotifScanOutput/KmeansCluster5/homerResults/motif16.similar.html]

motif16

## Information for motif16

A
G
T
C
A
G
T
C
A
G
T
C
A
G
T
C
C
T
G
A
A
C
G
T
A
C
T
G
C
G
T
A
A
C
G
T
A
G
T
C
A
G
T
C
C
G
T
A
C
T
G
A
A
C
G
T
  
Reverse Opposite:  

C
G
T
A
A
G
C
T
A
C
G
T
A
C
T
G
C
T
A
G
C
G
T
A
A
C
G
T
A
G
T
C
C
G
T
A
A
G
C
T
A
C
T
G
A
C
T
G
A
C
T
G
A
C
T
G
  

|  |  |
| --- | --- |
| p-value: | 1e-59 |
| log p-value: | -1.375e+02 |
| Information Content per bp: | 1.935 |
| Number of Target Sequences with motif | 122.0 |
| Percentage of Target Sequences with motif | 3.07% |
| Number of Background Sequences with motif | 138.2 |
| Percentage of Background Sequences with motif | 0.32% |
| Average Position of motif in Targets | 104.5 +/- 77.0bp |
| Average Position of motif in Background | 112.2 +/- 67.1bp |
| Strand Bias (log2 ratio + to - strand density) | 0.1 |
| Multiplicity (# of sites on avg that occur together) | 1.00 |
| Motif File: | file (matrix) reverse opposite |

### Similar de novo motifs found

|  |  |  |  |  |  |  |  |
| --- | --- | --- | --- | --- | --- | --- | --- |
| Rank | Match Score | Redundant Motif | P-value | log P-value | % of Targets | % of Background | Motif file |
| 1 | 0.950 | A T G C A G T C G A T C C T G A G C A T C T A G T C G A G C A T G A T C G T A C G T C A T C G A C G A T | 1e-59 | -137.455818 | 3.72% | 0.54% | motif file (matrix) |
| 2 | 0.797 | A G T C A G T C T G A C G A T C A G T C C T G A A G C T C A T G T G C A A C G T | 1e-45 | -104.084554 | 3.87% | 0.83% | motif file (matrix) |
| 3 | 0.906 | G T A C A G T C G T A C A G T C C T G A A G C T C A T G C T G A A G C T A G C T A G T C C T G A | 1e-39 | -91.733242 | 2.16% | 0.25% | motif file (matrix) |
| 4 | 0.829 | C G T A A C G T A G T C C T G A A G C T A C T G A C T G A C T G A C T G | 1e-36 | -83.967769 | 2.34% | 0.35% | motif file (matrix) |
